# Zinc Intake and Status and Risk of Type 2 Diabetes Mellitus: A Systematic Review and Meta-Analysis

**DOI:** 10.3390/nu11051027

**Published:** 2019-05-08

**Authors:** José C. Fernández-Cao, Marisol Warthon-Medina, Victoria H. Moran, Victoria Arija, Carlos Doepking, Lluis Serra-Majem, Nicola M. Lowe

**Affiliations:** 1 Department of Nutrition and Dietetics, Faculty of Heath Sciences, University of Atacama, Av. Copayapu 2862, Copiapó, 1530000 Atacama Region, Chile; carlos.doepking@uda.cl; 2 Nutrition and Public Health, Universitat Rovira i Virgili, C/ Sant Llorenç 21, Reus, 43201 Tarragona, Spain; 3 Food Databanks National Capability, Quadram Institute Bioscience, Norwich Research Park, Norwich, Norfolk NR4 7UA, UK; marisol@warthon-medina.com; 4 International Institute of Nutritional Sciences and Food Safety Studies, University of Central Lancashire, Darwin Building c/o Psychology Scholl Office, Preston, Lancashire PR1 2HE, UK; NMLowe@uclan.ac.uk; 5 Maternal and Infant Nutrition and Nurture Unit, University of Central Lancashire, Preston, Lancashire PR 1 2HE, UK; VLMoran@uclan.ac.uk; 6 Research Group in Nutrition and Mental Health (NUTRISAM), Institut d’Investigació Sanitària Pere Virgili (IISPV), Rovira i Virgili University, Reus, 43201 Tarragona, Spain; victoria.arija@urv.cat; 7 Research Institute of Biomedical and Health Sciences, University of Las Palmas de Gran Canaria and CHUIMI, Canarian Health Service, 35016 Las Palmas de Gran Canaria, Spain; lluis.serra@ulpgc.es; 8 Consorcio CIBER, M.P. Fisiopatología de la Obesidad y Nutrición (CIBERObn), Instituto de Salud Carlos III (ISCIII), 28029 Madrid, Spain

**Keywords:** zinc intake, zinc status, trace elements, type 2 diabetes mellitus, systematic review, meta-analysis, epidemiology

## Abstract

Zinc could have a protective role against type 2 diabetes mellitus (T2DM). This systematic review and meta-analysis aimed to evaluate the association between dietary, supplementary, and total zinc intake, as well as serum/plasma and whole blood zinc concentration, and risk of T2DM. Observational studies, conducted on cases of incident diabetes or T2DM patients and healthy subjects that reported a measure of association between zinc exposure and T2DM, were selected. Random effects meta-analyses were applied to obtain combined results. Stratified meta-analyses and meta-regressions were executed to assess sources of heterogeneity, as well as the impact of covariates on the findings. From 12,136 publications, 16 studies were selected. The odds ratio (OR) for T2DM comparing the highest versus lowest zinc intake from diet was 0.87 (95% CI: 0.78–0.98). Nevertheless, no association between supplementary or total zinc intake from both diet and supplementation, and T2DM was observed. A direct relationship was found between serum/plasma zinc levels and T2DM (OR = 1.64, 95% CI: 1.25–2.14). A moderately high dietary zinc intake, in relation to the Dietary Reference Intake, could reduce by 13% the risk of T2DM, and up to 41% in rural areas. Conversely, elevated serum/plasma zinc concentration was associated with an increased risk of T2DM by 64%, suggesting disturbances in zinc homeostasis.

## 1. Introduction

Diabetes mellitus is a major public health challenge worldwide, and is a key contributor to morbidity and mortality. In 2016, diabetes mellitus was listed as the seventh leading cause of death globally [1]. According to the International Diabetes Federation (IDF) Diabetes ATLAS, the global prevalence of diabetes among individuals aged 20–79 years in 2017 was 8.8% (95% confidence interval (CI): 7.2–11.3), i.e., 424.9 million people, with a total healthcare expenditure estimated at just under USD 727 billion [2]. The number of people suffering from diabetes is expected to increase to 628.6 million in 2045, a prevalence of over 9.9% (95% CI: 7.5–12.7). Around 90% of cases of diabetes are type 2 diabetes (T2DM) [2]. This disease results from the body’s ineffective use of insulin [1], and is the result of the interaction of multiple genetic and environmental factors [3].

The role of zinc in the etiology of T2DM has been widely reported in recent decades. Longitudinal large prospective cohort studies, such as the Nurses’ Health Study (NHS) cohort [4] in the USA; the Australian Longitudinal Study on Women’s Health cohort study [5]; the Malmö Diet and Cancer Study cohort [6] in Sweden; and the Japan Collaborative Cohort study [7], among others, have investigated the effect of dietary, supplementary, and/or total zinc intake on the risk of developing T2DM. The NHS cohort was the first to prospectively analyze these relationships, and it reported that the higher the total and/or dietary zinc intake, the lower the risk of T2DM over subsequent years [4]. Although a non-significant association was observed between supplementary zinc intake and risk of T2DM in the overall sample, an inverse relationship was seen in those participants with low dietary zinc intake [4]. There is currently no evidence that supports the use of zinc supplements in the prevention of T2DM [8]. Nevertheless, a recent clinical trial based on zinc supplementation has found a reduction in the progression to diabetes in prediabetic subjects [9]. Some subsequent prospective cohort studies, however, have failed to confirm some of the results reported in the NHS cohort [6,10,11,12]. A systematic review of prospective studies that aimed to examine the role of zinc intake and status on the risk of T2DM revealed inconsistencies between studies, and suggested the possible influence of confounding factors on these relationships [13].

Similarly, findings on the relationship between serum/plasma zinc concentration and T2DM are contradictory [14,15,16]. The prospective Kuopio Ischaemic Heart Disease Risk Factor (KIHD) cohort study of 2220 Finnish men followed over twenty years showed that higher serum zinc levels were associated with an increased risk of T2DM [14]. Conversely, a cross-sectional study of 128 Russian postmenopausal women found an inverse relationship between serum zinc and T2DM [17]. The relationship between whole blood zinc concentration and T2DM has been investigated by two studies carried out within the same population-based Nord-Trøndelag Health Study (HUNT3), but their results were inconclusive [18,19]. The study conducted on newly diagnosed T2DM patients found a positive association between whole blood zinc concentration and T2DM [18], while the study performed in previously diagnosed T2DM patients showed no association [19]. In our previous systematic review and meta-analysis, which aimed to compare whole blood zinc concentration between T2DM patients and non-diabetic subjects, we observed a lower whole blood zinc concentration in T2DM patients [20]. It should be noted that diabetic subjects had, at least, 10.2 ± 8.6 years of duration of diabetes. Therefore, the duration of diabetes may have an impact on this association, and it is important to clarify this relationship.

The mechanism whereby zinc could have an impact on the risk of T2DM has not been completely elucidated, however zinc is an essential trace element that is involved in the physiology of carbohydrate metabolism in many ways. Zinc participates in the adequate insulin synthesis, storage, crystallization, and secretion in the pancreatic β-cell, as well as action and translocation of insulin into the cells [21,22,23,24]. In addition, zinc seems to play a role in insulin sensitivity through the activation of the phosphoinositol-3-kinase/protein kinase B cascade [25]. Due to its insulin–mimetic action, zinc also stimulates glucose uptake in insulin-dependent tissues [26]. Moreover, zinc is implicated in the suppression of proinflammatory cytokines, such as interleukin-1β [27] and nuclear factor kβ [28], avoiding β-cells’ death and protecting insulin. All of these functions of zinc could support its potential protective role against diabetes mellitus.

Much remains uncertain concerning the effect of zinc on the risk of developing T2DM. Therefore, the purpose of this comprehensive systematic review and meta-analysis of observational studies was to evaluate the association between dietary, supplementary, and total zinc intake, as well as serum/plasma zinc and whole blood concentration and risk of T2DM in the adult population. A secondary objective was to examine potential confounding factors that may impact on these relationships.

## 2. Materials and Methods

The protocol for this systematic review and meta-analysis of observational studies was registered in PROSPERO (2015: CRD42015020178) and can be accessed here: (http://www.crd.york.ac.uk/PROSPERO/display_record.php?ID=CRD42015020178). The study was conducted in accordance with the Meta-Analyses of Observational Studies in Epidemiology (MOOSE) criteria statement [29]. The MOOSE checklist is shown in Supplementary Materials Table S1.

### 2.1. Search Strategy

**Figure 1:**
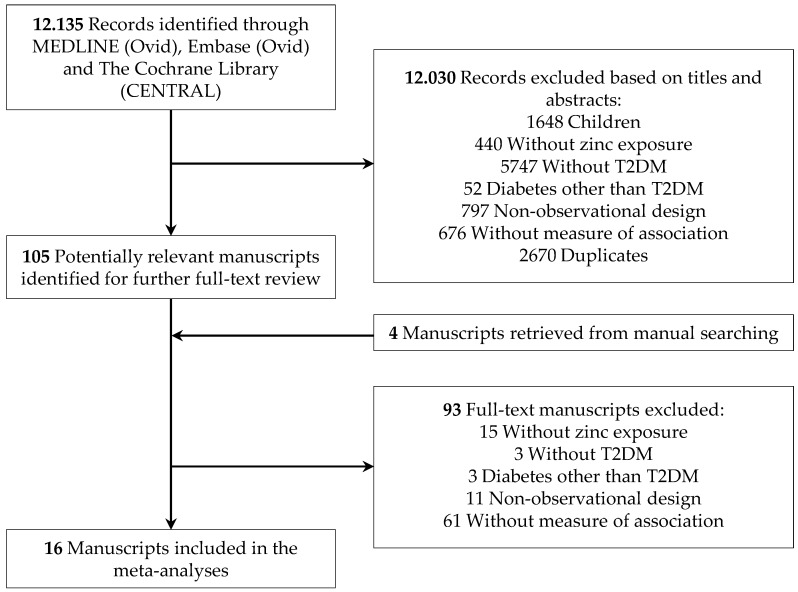
Flowchart of the selection process.

This systematic review and meta-analysis were carried out by six investigators within the framework of the EURopean micronutrient RECommendations Aligned (EURRECA) Network of Excellence, one aim of which was to undertake a series of systematic search for studies assessing the effect of zinc on different health outcomes. A comprehensive search was developed in MEDLINE (Ovid), Embase (Ovid), and The Cochrane Library (CENTRAL) up to January 2019, using search terms for (“study designs in humans”) AND (Zinc) AND (intake OR status). Additional articles were identified through manual searching and citation tracking (Figure 1).

### 2.2. Study Eligibility Criteria

Studies were selected according to the following inclusion criteria: (1) studies of observational design, including prospective cohort, case-control, and cross-sectional; (2) studies conducted on human adults, with type 2 diabetes mellitus (T2DM) or cases of incident diabetes and healthy control individuals or controls of non-incident diabetes; (3) publications reported in English, Spanish or other European languages; (4) studies that reported a measure of association, such as relative risk (RR), odds ratio (OD) or hazard ratio (HR), between dietary, supplementary, and/or total zinc intake and/or serum/plasma and/or whole blood zinc concentration and T2DM, through a multivariable adjusted analysis that compared the highest quantile of zinc exposure versus the lowest. Studies that compared user versus non-user of zinc supplements in relation to T2DM were also selected. Other kinds of observational study designs, such as case reports, case series or ecological studies; reviews; and experimental or quasi-experimental studies, as well as those with participants diagnosed with diabetes mellitus other than T2DM, were excluded.

### 2.3. Study Selection

Titles and abstracts of studies identified through the literature search were independently screened for eligibility. Subsequently, the full text of relevant studies was retrieved and examined further against the inclusion and exclusion criteria. Reasons for excluding studies were recorded. The selection process was independently completed by members of the research team (JCFC, MWM, VHM, CD, and NL). A 10% sample was cross-checked by another investigator (MWM) to ensure consistency between reviewers, and any discrepancy or disagreement was resolved by discussion until consensus was reached among the reviewers.

### 2.4. Data Extraction and Study Quality Assessment

One reviewer (JCFC) carried out the data extraction process using a data-extraction spreadsheet. Two other reviewers (VHM and NL) independently screened the accuracy of the extracted data. In order to avoid the inclusion of duplicate data in the meta-analyses, some strategies were applied: first, the name of the project was recorded for all studies that met the inclusion criteria, as well as the geographic location where the studies had been conducted; second, the lists of authors were compared among them. Complementary data from the same project was included for a qualitative summary.

From each manuscript selected for inclusion, the following data were extracted into an excel spreadsheet: study identification (first author’s name, year of publication, and name of the project), study characteristics (study design, period of follow-up, measure of association, adjustment variables, quality score, country, geographic regions, geographic area, sample base, matched design, sample size for each group and total, zinc assessment method, zinc quantiles adjusted for energy, ascertainment of T2DM, percentage of T2DM subjects, effect size, and 95% confidence interval (CI) for the most adjusted model), and study population (age, gender, ethnicity, area of residence—dietary, supplementary and total zinc intake, as well as serum, plasma, and whole blood zinc concentration—BMI, fasting glucose levels, stage of diabetes). To incorporate relevant data in forms other than the mean and standard deviation, such as median and the interquartile range, estimation methods proposed by Wan et al., [24] were applied, which are valid for both normal and skewed data. When covariates of interest were expressed as a range, the midpoint of the range was assumed. If any of the data were missing, the authors were contacted for additional data.

The quality of studies selected was evaluated by one research investigator (JCFC) using the Strengthening the Reporting of Observational Studies in Epidemiology (STROBE) Statement [25]. The STROBE checklist is shown in Supplementary Materials Table S2. The quality score was used to assess its possible influence on results.

### 2.5. Statistical Analysis

Meta-analyses comparing the highest versus the lowest quantile of exposure to zinc intake and/or status were performed when at least two studies with a common exposure in relation to T2DM were available. For all meta-analyses, effect size and 95% CIs were log-transformed. Estimated standard errors were calculated from log 95% CIs by subtracting the lower bound of the CI from the upper bound and subsequently dividing by two times 1.96. The method of a random-effects model and the generic inverse variance method were used to calculate the pooled effect sizes, reported as OR and 95% CI. Relative risks and hazard ratios were deemed equivalent to ORs [30]. The most adjusted model of the multivariable analysis in the selected studies was used to estimate the effect size in all meta-analyses. Forest plots were created to visualize individual and global estimates. As the studies included in the meta-analysis on supplementary zinc intake and T2DM reported exposure either in quantiles or as dichotomous variable (user versus no user), a stratified meta-analysis was performed based on these criteria.

Univariate and multivariate meta-regressions with Knapp–Hartung modification [31] were conducted to examine the potential impact of certain covariates on effect size. To display relevant results of a single continuous covariate in univariate meta-regressions, bubble plots were created. This graph represents the fitted regression line together with circles representing the estimates from each study, sized according to the precision of each estimate (the inverse of its within-study variance). Multivariate meta-regressions models were executed adding the covariate with the strongest association in univariate analysis first and then adding the next one in turn. Covariates showing collinearity were removed from the final multivariate model. Finally, a meta-regression equation was generated using the intercept (a), as well as the regression coefficient (b) of a specific covariate, to know how the effect size (OR for T2DM) changes with a unit increase in the exploratory covariate (Ln (OR_T2DM_) = (a) + b × (covariate)).

Heterogeneity was assessed by the Cochran Q-statistic and the I^2^ statistic to quantify the percentage of variation attributable to between-study heterogeneity [32]. I^2^ values of 25%, 50%, and 75% were considered as low, medium, and high heterogeneity, respectively [33,34]. Potential sources of heterogeneity were explored through stratified analyses and univariate meta-regressions, even if an initial heterogeneity was non-significant [35], using different variables. Thus, categorical variables were: study design, study design and area of residence, measures of association, quality score, geographic regions, location, sample base, matched design, sample size, zinc intake assessment method, zinc serum/plasma assessment method, ascertainment of T2DM, diagnostic pattern, percentage of T2DM, gender, ethnicity, area of residence, group with higher serum/plasma zinc levels, zinc quantiles adjusted for energy. In addition, continuous variables were also used, such as sample size for each group and total, period of follow-up (years), quality score (%), percentage of T2DM subjects (%), age in cases and controls (years), age difference and ratio between cases and controls (years), serum/plasma zinc levels in cases and controls (µg/dL), serum/plasma zinc difference and ratio between cases and controls (µg/dL), BMI in cases and controls (kg/m^2^), BMI difference and ratio between cases and controls (kg/m^2^), fasting glucose levels in cases and controls (mmol/L), and fasting glucose difference and ratio between cases and controls (mmol/L). Multivariate meta-regressions were also utilized to examine further the covariates that had a significant influence on heterogeneity in univariate analysis. In addition, the proportion of between-study variance explained by one or more covariates was estimated through the adjusted R^2^ (R_A_^2^). Likewise, the percentage of residual variation due to heterogeneity which remains unexplained by one or more covariates (I_r_^2^) was obtained.

To assess the power of each study on the overall pooled estimates, sensitivity analysis was performed using the leave-one-out method [36], where one study was excluded at a time, evaluating the impact of removing each of the studies on the summary results and the between-study heterogeneity. Furthermore, publication bias was investigated by visual inspection of funnel plots and quantitatively assessed using Egger’s [37] and Begg’s [38] tests. All analyses were performed with STATA statistical software, version 15.0. (STATA Corp., College Station, Texas, USA).

## 3. Results

The literature search strategy generated 12,136 publications, and 16 studies were finally selected for this systematic review and meta-analysis of observational studies [4,5,6,7,10,11,12,14,15,16,17,18,19,39,40,41]. There were no studies that were excluded for reasons of language. The details of the selection process and the reasons for exclusion are shown in the flowchart (Figure 1). The quality of selected publications, according to the Strengthening the Reporting of Observational Studies in Epidemiology (STROBE) Statement [42], was high. The compliance percentages of the STROBE items were between 69 and 100%, 14 of the 16 selected studies above 80% [4,5,6,7,10,11,12,14,15,16,17,18,19,39,41]. The characteristics of the included studies for meta-analyses are summarized in Table 1, Table 2 and Table 3.

### 3.1. Dietary Zinc Intake and T2DM

Seven prospective cohort studies [4,5,6,7,10,11] and one cross-sectional study [39] were included in the meta-analysis of the association between dietary zinc intake and T2DM (Table 1). Five studies were carried out in the western countries (USA, the Nurses’ Health Study (NHS) cohort [4], the Multi-Ethnic Study of Atherosclerosis (MESA) cohort [10], and the Coronary Artery Risk Development in Young Adults (CARDIA) cohort [11]; Australia, the Australian Longitudinal Study on Women’s Health (ALSWH) cohort [5]; and Sweden, the Malmö Diet and Cancer Study (MDCS) cohort [6]), and two in the eastern countries (India [39], and Japan, the Japan Collaborative Cohort (JACC) [7]). This meta-analysis comprised 146,027 participants aged between 18 and 84 years, and of both genders, belonging to different ethnic groups (Hispanic, Caucasian, African American, Chinese or South Asian, among others), and areas of residence (rural or urban). During the follow-up of participants, between 4.8 years on average in the MESA cohort [10] and 24 years in the NHS cohort [4], 11,511 cases of T2DM were detected (7.8%). The percentage of T2DM cases was highly variable between the studies, from 2.5% in the JACC study [7] to 14.1% in the Swedish MDCS cohort [6].

Dietary zinc intake was collected using validated food frequency questionnaires (VFFQs) [4,5,7,10], validated diet history questionnaires (VDHQ) [6,11], or a 7-day dietary record [39]. The mean of dietary zinc intake ranged from 5.6 ± 1.6 mg/day in urban women from India [39] to 16.7 mg/day in urban subjects from the USA [11]. Ascertainment of T2DM was carried out through different criteria (fasting plasma glucose (FPG) and/or oral glucose tolerance test (OGTT), and/or self-reported, and/or using registries from different institutions, and/or use of antidiabetic drugs).

**Figure 2:**
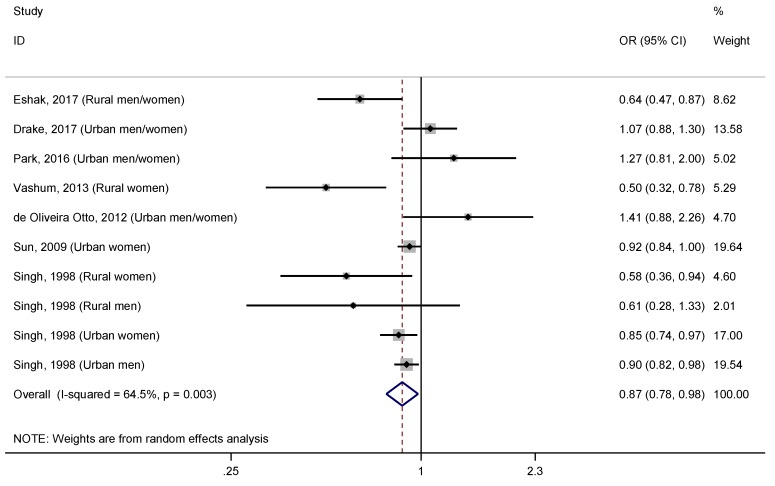
Forest plot of pooled effect size of the highest versus lowest dietary zinc intake for T2DM. Squares represent odds ratios (OR) for each study, and the size of the square is the study-specific statistical weight. Horizontal lines indicate the 95% CI of each study. Diamond represents the combined OR estimate with corresponding 95% CI.

To evaluate the association between the dietary zinc intake and the T2DM, a meta-analysis was conducted (Figure 2). The pooled effect size for T2DM comparing the highest versus lowest dietary zinc intakes was 0.87 (95% CI: 0.78–0.98), with moderate to high heterogeneity (I^2^ = 64.5%, *p* = 0.003).

**Figure 3:**
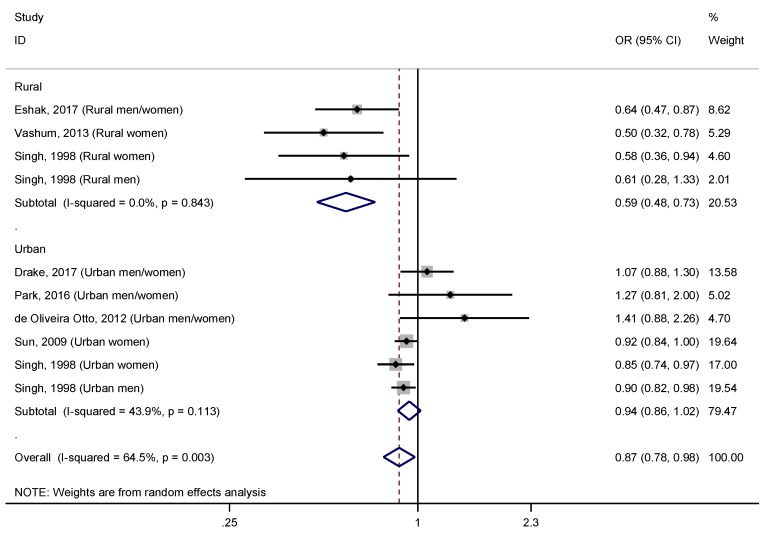
Forest plot of pooled effect size of the highest vs. lowest dietary zinc intake for T2DM according to area of residence (rural vs. urban). Squares represent ORs for each study, and the size of the square is the study-specific statistical weight. Horizontal lines indicate the 95% CI of each study. Diamond represents the combined OR estimate with corresponding 95% CI.

**Figure 4:**
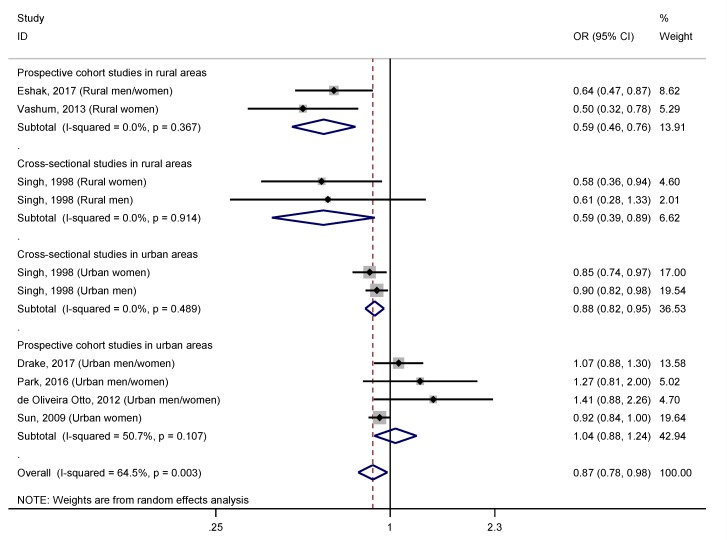
Forest plot of pooled effect size of the highest vs. lowest dietary zinc intake for T2DM according to study design and area of residence (prospective cohort studies in rural areas, cross-sectional studies in rural areas, cross-sectional studies in urban areas, and prospective cohort studies in urban areas). Squares represent ORs for each study, and the size of the square is the study-specific statistical weight. Horizontal lines indicate the 95% CI of each study. Diamond represents the combined OR estimate with corresponding 95% CI.

Through a stratified analysis based on the area of residence of participants, rural versus urban, (Figure 3) we observed a higher and significant effect size in rural areas (OR = 0.59, 95% CI: 0.48–0.73), and undetectable heterogeneity (I^2^ = 0.0%, *p* = 0.843), meanwhile in urban areas the effect became non-significant (OR = 0.94, 95% CI: 0.86–1.02; I^2^ = 43.9%, *p* = 0.113). Subsequently, we used the adjusted R^2^ to examine how much of the heterogeneity was accounted for by the area of residence (Table 4), and we found that the heterogeneity was explained to a great extent (R_A_^2^ = 100.0%; I_r_^2^ = 17.8%). Interestingly, through a stratified analysis by the covariate “study design and area of residence”, we observed that this protective effect of dietary zinc intake in rural areas was found in both cross-sectional and prospective studies (Figure 4). In this analysis, results were statistically significant, and heterogeneity was reduced (I^2^ = 0.0%, *p* > 0.050) for all subgroups, except for prospective studies conducted in urban areas (OR = 1.04, 95% CI: 0.88–1.24; I^2^ = 50.7%, *p* = 0.107).

**Figure 5:**
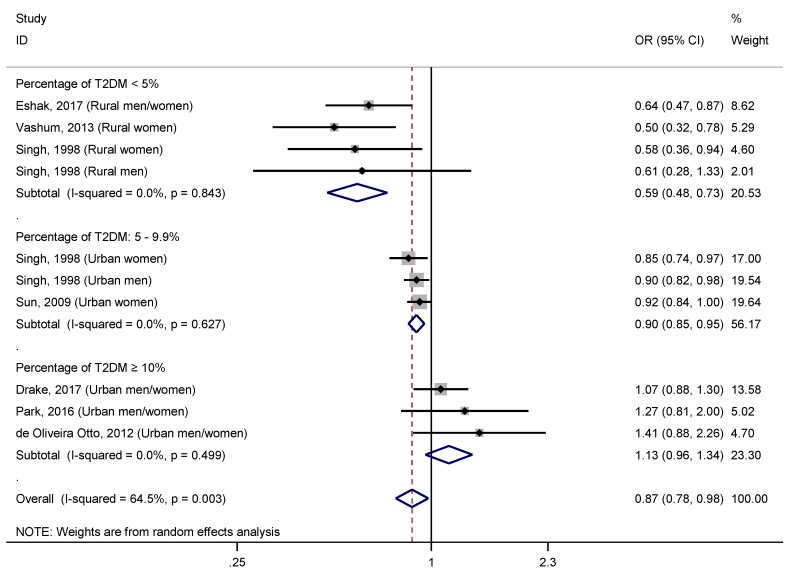
Forest plot of pooled effect size of the highest vs. lowest dietary zinc intake for T2DM according to the percentage of T2DM (<5%/5–9.9%/≥10%). Squares represent ORs for each study, and the size of the square is the study-specific statistical weight. Horizontal lines indicate the 95% CI of each study. Diamond represents the combined OR estimate with corresponding 95% CI.

The corresponding adjusted R^2^ for this covariate was 90.47%. However, the covariate that showed the greatest impact on the relationship studied was the percentage of T2DM, both as continuous and categorized variable. Thus, the stratified analysis (Figure 5) by this covariate categorized (<5/5–9.9/≥10) revealed a significant protective effect of dietary zinc intake in those studies with <5% of T2DM (OR = 0.59, 95% CI: 0.48–0.73; I^2^ = 0.0%, *p* = 0.843), and those between 5–9.9% (OR = 0.90, 95% CI: 0.85–0.95; I^2^ = 0.0%, *p* = 0.627), but not when it was higher than 10% (OR = 1.13, 95% CI: 0.96–1.34; I^2^ = 0.0%, *p* = 0.499). It should be noted that the level of heterogeneity was reduced to 0.0% in all these subgroups. The importance of this covariate in the assessed relationship was supported by the large proportion of the between-study variance was explained (R_A_^2^ = 100.0%), as well as the undetectable percentage of the residual variation that was attributable to the between-study heterogeneity, after entering this covariate into a univariate meta-regression model (I_r_^2^ = 0.0%).

**Figure 6:**
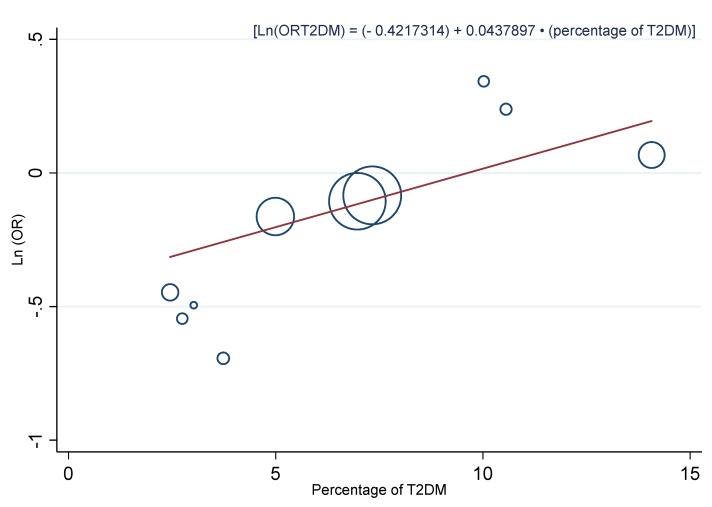
Bubble plot with a fitted meta-regression line of the relationship between the Ln(OR) and the percentage of T2DM. Circles are sized according to the precision of each estimate (the inverse of its within-study variance).

A multivariate meta-regression model adding the three covariates with a significantly higher impact on the association, showed that only a “percentage of T2DM” continued being significant (0.23, 95% CI: 0.02, 0.45, *p* = 0.037). Once the fourth covariate was introduced, none remained significant. When we analyzed the percentage of T2DM as a continuous variable, similar results were observed (R_A_^2^ = 100.0%, I_r_^2^ = 23.6%). A bubble plot was used to represent this covariate (Figure 6), and it was found that, as the percentage of T2DM increased, the protective effect of a moderately high dietary zinc intake was reduced in a relationship defined by the equation of the regression line: (Ln(OR_T2DM_) = (−0.4217314) + 0.0437897 × (percentage of T2DM)).

The effect size ranged between (OR = 0.84, 95% CI: 0.75–0.96) after excluding the study carried out by Drake et al. [6], and (OR = 0.90, 95% CI: 0.81–0.99), after excluding the study conducted by Vashum et al. [5]. However, the combined overall effect size remained on the verge of statistical significance after removing the data obtained by Singh et al. [39] in urban Indian women (OR = 0.87, 95% CI: 0.76–1.00), or that of Eshak et al. [7] in Japanese subjects (OR = 0.90, 95% CI: 0.80–1.01). Finally, for this meta-analysis, an overall symmetry of the funnel plots was observed by visual inspection (Supplementary Materials Figure S1). This was confirmed by the Egger’s (*p* = 0.429) and Begg’s (*p* = 0.721) tests, indicating the absence of publication bias.

### 3.2. Supplementary Zinc Intake and T2DM

Three studies of 313,003 individuals assessed the association between supplementary zinc intake and T2DM (NHS cohort [4], National Institutes of Health-American Association of Retired Persons (NIH-AARP) Diet and Health Study [12], MDCS cohort [6]) (Table 2). The follow-up period of these prospective cohort studies ranged between 10 [12] to 24 years [4]. Participants were white (mostly) women [4] or subjects of both genders [6,12], aged between 33 and 71 years, and from urban areas. In total, 17,806 patients with incident diabetes (between 6.1 and 14.1%) were identified according to different diagnostic criteria (self-reported [4,12], or through an FPG ≥ 7.0 mmol/L measured twice, and institutional registries [6]).

Supplementary zinc intake was determined using different food intake instruments, such as VFFQ [4], VDHQ [6], or dietary survey, including an FFQ and a short survey [12]. The percentage of patients with incident diabetes supplemented with zinc was around 12.5% [6,12], meanwhile in controls of non-incident diabetes, zinc supplementation ranged between 5.7% in the NIH-AARP Diet and Health Study [12], to 17.7%, in the MDCS cohort [6]. The NHS cohort reported a large increase in the proportion of women who were supplemented with zinc in 2004 (48.6%) compared with 1980 (6.3%) [4].

**Figure 7:**
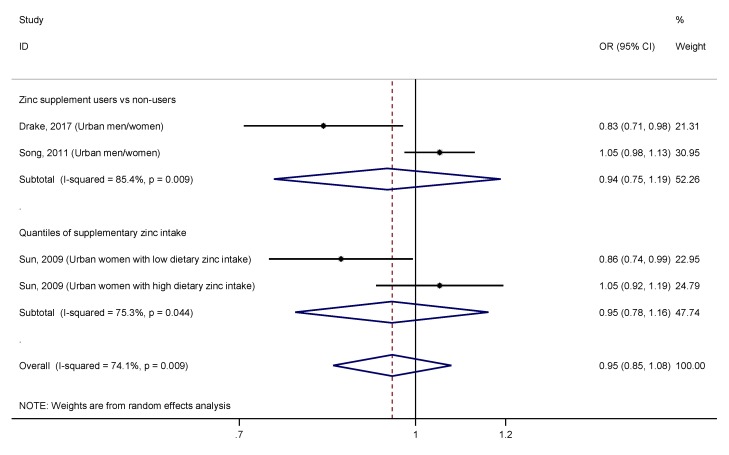
Forest plot of the pooled effect size of the highest versus lowest supplementary zinc intake for T2DM according to the analysis (zinc supplement users/non-users versus quantiles of supplementary zinc intake). Squares represent ORs for each study, and the size of the square is the study-specific statistical weight. Horizontal lines indicate the 95% CI of each study. Diamond represents the combined OR estimate with corresponding 95% CI.

The association between supplementary zinc intake and the risk of T2DM was evaluated through a meta-analysis stratified by whether the analysis had been done comparing zinc supplement users versus non-users or comparing the highest versus lowest quantile of supplementary zinc intake, in order not to introduce bias in the analysis (Figure 7). Results revealed a non-significant association between zinc supplement users versus non-users and T2DM (OR = 0.94, 95% CI: 0.75–1.19; I^2^ = 85.4%, *p* = 0.009), and between higher supplementary zinc intake versus lower and T2DM (OR = 0.95, 95% CI: 0.78–1.16; I^2^ = 75.3%, *p* = 0.044), and an elevated heterogeneity in both cases.

Publication bias was unlikely in this meta-analysis, according to Egger’s (*p* = 0.186), and Begg’s (*p* = 0.089) tests (Figure S2).

### 3.3. Total Zinc Intake and T2DM

The final data set for the meta-analysis of total zinc intake and risk of T2DM included only two large prospective cohort studies [4,6] (Table 2). Nevertheless, both studies comprised 108,429 individuals, 9706 patients with incident diabetes and 98,723 controls of non-incident diabetes. Incidence of T2DM was 14.1% in the middle-aged Swedish cohort of urban men and women (MDCS) [6], and 7.3% in the American cohort of urban women (NHS) [4]. A VFFQ [4] and a VDHQ [6] were used to determine the total zinc intake, that ranged from 4.9 to 18.0 mg/day in the NHS cohort [4], and around 12.9 ± 5.4 mg/day in controls of non-incident diabetes and 13.0 ± 6.2 mg/day in patients with incident diabetes from the MDCS cohort [6].

**Figure 8:**
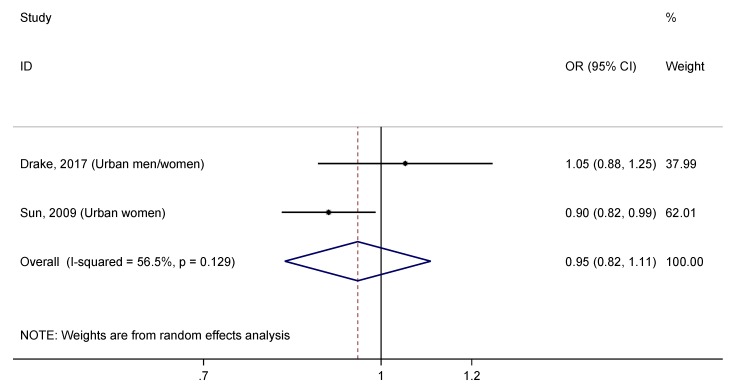
Forest plot of pooled effect size of the highest vs. lowest total zinc intake for T2DM. Squares represent ORs for each study, and the size of the square is the study-specific statistical weight. Horizontal lines indicate the 95% CI of each study. Diamond represents the combined OR estimate with corresponding 95% CI.

After pooling data from both studies (Figure 8), we found that total zinc intake was not significantly associated with the incidence of T2DM (OR = 0.95, 95% CI: 0.82–1.11). There was moderate heterogeneity between the studies (I^2^ = 56.5%, *p* = 0.129). Moreover, no evidence of publication bias was found (*p* = 1.000) (Supplementary Materials Figure S3).

### 3.4. Whole Blood Zinc Concentration and T2DM

Only two cross-sectional studies carried out within the third survey of the population-based Nord-Trøndelag Health Study (HUNT3 Survey) were identified to assess the relationship between concentration of zinc in whole blood and T2DM [18,19]. The fact that both studies shared part of the same study sample prevented the execution of a meta-analysis to obtain a pooled result. Nevertheless, a qualitative summary was conducted to present the findings of these two studies (Table 3). Participants in both studies were men and women, mainly Caucasian, and aged around 61.5 ± 8.7 years old, who lived mainly in rural areas from Norway. The main difference between these two studies was the sampling strategy. Hansen et al. [18] selected 876 subjects at high risk for T2DM, but without previously known diabetes. In this study, 128 previously undiagnosed cases of T2DM, were detected by screening [18]. In contrast, Simic et al. [19] included 883 subjects, of which 267 had self-reported T2DM, i.e., they were patients in a more advanced stage of the disease. Curiously, while Hansen et al. [18] found a significant and positive association between a higher whole blood zinc concentration and the onset of T2DM (OR = 2.19, 95% CI: 1.05–4.59), Simic et al. [19] did not observe any significant relationship (OR = 1.08, 95% CI: 0.59–1.97). Differences in concentrations of zinc in whole blood between cases and controls, measured in both studies through inductively coupled plasma mass spectrometry (ICP-MS), were more evident in the study conducted by Hansen et al. [18] (median in cases: 799.0 µg/dL; median in controls: 754.0 µg/dL) compared to the one carried out by Simic et al. [19] (median in cases: 764.3 µg/dL; median in controls: 751.2 µg/dL). It is worth noting that the median whole blood zinc concentration in the control subjects in both studies was very similar, but not in those of the diabetic subjects.

### 3.5. Serum/Plasma Zinc Concentration and T2DM

Six observational studies (one prospective cohort study [14], one nested case-control study [15], and four cross-sectional studies [16,17,40,41]) were included in the meta-analysis of serum/plasma zinc concentration and T2DM (Table 3). Four studies were carried out on Chinese urban men and women between the ages of 40 and 90 years [15,16,40,41], one in Russian women with an average age of 56.3 ± 5.7 years [17], and one in Finnish men aged between 42 and 60 years [14]. The total number of cases of T2DM was 2936, among 8392 participants. The period of follow-up was between 4.6 years in the nested case control within the Dongfeng–Tongji (DFTJ) cohort [15], and 20 years in the KIHD study [14]. Serum/plasma zinc concentration was determined mainly by ICP-MS [15,16,17,40,41], meanwhile the KIHD used atomic absorption spectrophotometry (AAS) [14]. The levels of serum/plasma zinc in controls ranged from a median of 57.5 µg/dL to a mean of 172.5 ± 73.0 µg/dL; and in cases from a median of 63.4 µg/dL to a mean of 169.6 ± 142.4 µg/dL.

**Figure 9:**
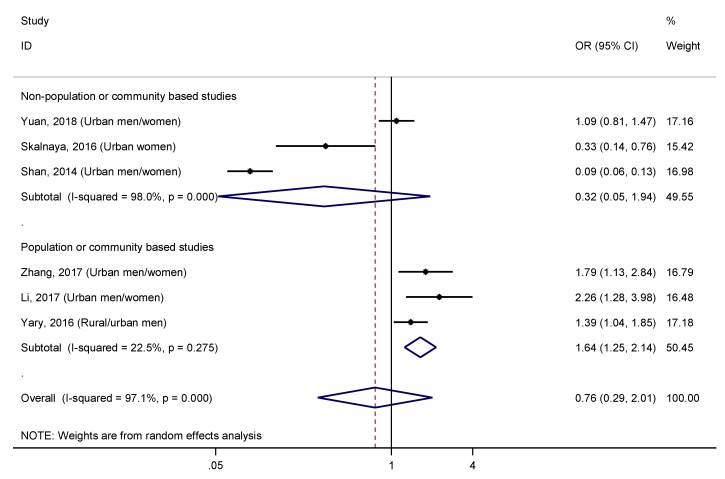
Forest plot of pooled effect size of the highest vs. lowest serum/plasma zinc concentration for T2DM according to the sample base (population/community-based studies vs. non-population/community-based studies). Squares represent ORs for each study, and the size of the square is the study-specific statistical weight. Horizontal lines indicate the 95% CI of each study. Diamond represents the combined OR estimate with corresponding 95% CI.

The combined effect size of T2DM for the highest versus lowest quantile of serum/plasma zinc concentration was 0.76 (95% CI: 0.29–2.01). However, a high level of heterogeneity was found (I^2^ = 97.1%, *p* < 0.001). Sensitivity analysis omitting one study at a time and calculating the heterogeneity for the remainder of the studies showed that the study conducted by Shan et al. [16] substantially influenced the overall heterogeneity, resulting in a reduction around 31% of this when it was excluded. In addition, the elimination of this study and the one carried out by Skalnaya et al. [17], decreased the level of heterogeneity by 44.5%, showing their impact on results (OR = 1.47, 95% CI: 1.11–1.95; I^2^ = 53.9%, *p* = 0.090). Nevertheless, the exclusion of any other study had a negligible effect on heterogeneity. When performing stratified analysis for “sample base” (Figure 9), a significant and positive association was found in the subgroup of “population or community-based studies” (OR = 1.64, 95% CI: 1.25–2.14), and a low heterogeneity (I^2^ > 22.5%, *p* = 0.275). On the other hand, “non-population or community-based studies” showed a very high level of heterogeneity (I^2^ > 98.0%, *p* < 0.001). Likewise, when it was stratified by the covariate “group with higher zinc levels”, a relationship between serum/plasma zinc levels and T2DM (OR = 1.47, 95% CI: 1.11–1.95; I^2^ = 53.9%, *p* = 0.090) was observed in the subgroup of studies with higher zinc levels in the case group compared to controls (Table S3). Meanwhile, a significant negative relationship was found in the subgroup in which controls had higher serum/plasma concentration (OR = 0.16, 95% CI: 0.05–0.54), but with a high heterogeneity (I^2^ = 86.4%, *p* = 0.007). Finally, the difference in mean serum/plasma zinc concentration between cases and controls, as well its ratio, also explained, to a large extent, the heterogeneity observed (R_A_^2^ = 85.2%, for mean difference; and R_A_^2^ = 92.6%, for mean ratio), as expected.

Although the funnel plot showed some degree of asymmetry (Figure S3), we did not detect any risk of publication bias according to the Egger’s (*p* = 0.815) or Begg’s tests (*p* = 0.707).

## 4. Discussion

This systematic review and meta-analysis of observational studies found an inverse association between dietary zinc intake and T2DM. This could suggest a potential beneficial role of zinc from diet to prevent the risk of this disease. In addition, the relationship seemed to be more evident in rural areas, and when the proportion of T2DM cases in the population was low or moderate. Conversely, a non-significant association between total or supplementary zinc intake and T2DM was observed, although data are limited. Whole blood zinc concentration could be directly related to T2DM only at an early phase of the diabetes disease, as suggested by results from the same cohort study. This hypothesis could not be examined for serum/plasma zinc concentration. Nevertheless, a positive relationship was found between this biomarker of zinc status and T2DM in population-based studies.

Our results suggest that a diet moderately elevated in zinc could help to prevent the development of T2DM. We tried to determine the cut-off point or range of dietary zinc intake with a protective effect against type 2 diabetes mellitus (T2DM); however, data were imprecise, heterogeneous, and not reported in all the studies. Despite these limitations in the data of the selected studies, it was notable that when the lowest quantiles (reference) did not reach the dietary reference intakes (DRI) according to the Institute Of Medicine (IOM) for adult men (11 mg/day) and women (8 mg/day) [43], those quantiles of dietary zinc intake that reached or moderately exceeded the DRI showed a protective effect, even in the intermediate quantiles [4,5]. Furthermore, when the highest quantiles of dietary zinc intake did not reach the DRI, no significant association was observed [10]. Interestingly, when the lowest quantiles of dietary zinc intake reached the DRI, the highest quantiles (>23.34 mg/day) did not show a protective effect on T2DM, and could even have a harmful impact on the risk of T2DM as observed in a model not fully adjusted [11]. These data seem to suggest that a dietary zinc intake within or slightly above the DRI could have a protective role on the risk of T2DM, but not when intake is very high. Consistent with our findings, several observational studies have shown a protective effect of a moderately high dietary zinc intake on cardiometabolic conditions, such as metabolic syndrome [44,45] and gestational hyperglycemia [46], and mortality by cardiovascular disease [47] and all causes [48]. Conversely, other studies have found no significant [10,49], or even direct associations [50,51] between dietary zinc intake and some of these cardiometabolic events. The first systematic review of prospective cohort studies on the association between zinc status, including dietary zinc intake, and risk of cardiovascular disease and T2DM [13] revealed a limited number of studies on this topic, as well as the inconsistence of their results. As the authors themselves suggested, the effect of confounding factors may have played an important role in the observed findings. In our meta-analysis we have evaluated a large number of confounding factors in order to identify and quantify those that could impact on the relationship between dietary zinc intake and T2DM. Gender is one of the confounding factors most reported in the above mentioned studies on the relationship between dietary zinc intake and metabolic syndrome [44,45], cardiovascular disease [13], and mortality [47]. Our results showed a similar significant inverse association between dietary zinc intake and T2DM in both men and women, suggesting that gender does not seem to have a relevant role in this relationship.

Interestingly, we observed that the covariate “area of residence” of participants (rural versus urban) had a key effect on our findings. While a strong inverse association was observed in studies conducted on participants living in rural areas, a null relationship was observed in those studies carried out on urban subjects (Figure 3). Interestingly, when we addressed these findings also taking into account the design of the studies, we observed a 41% reduction in the risk of T2DM in both cross-sectional and prospective studies conducted in rural areas (Figure 4). Conversely, the effect size was reduced to 12% in cross-sectional studies performed in urban areas, and in prospective cohort studies, the association was not significant. These observations support the hypothesis that living in urban areas may counteract the beneficial effect of an elevated dietary zinc intake on risk of T2DM. Accumulating evidence strongly suggests that the change from rural to urban environments may have a marked impact on lifestyle [52,53], resulting in the increase of certain risk factors, such as unhealthy diets, sedentary behavior, and smoking, among others, that account for a large contribution to global burden of major disease [54,55]. Thus, it has been revealed that T2DM risk factors are more common in urban than in rural areas [56]. The greater exposure to risk factors in urban environments could explain the small or null protective effect of the intake of zinc from diet against the risk of T2DM. Indeed, it is known that there is higher prevalence of T2DM in urban compared to rural areas [2,56]. According to the International Diabetes Federation (IDF) Diabetes ATLAS edition 2017, the global prevalence of diabetes in urban areas was 10.2%, i.e., 279.2 million people aged between 20–79 years, meanwhile in rural areas was notably lower, 6.9% (145.7 million) [2]. In addition, the number of people living with diabetes in urban areas is expected to increase to 472.6 million in 2045, due mainly to global urbanization [2].

It is interesting that the covariate which had the greatest impact on the association between dietary zinc intake and T2DM was the proportion of T2DM cases identified in each study, both as a continuous and categorized variable. When we conducted a meta-regression introducing the percentage of T2DM as a continuous variable, we found that for each percentage point that increased this covariate, the protective effect of a moderately high dietary zinc intake, relative to the DRI, against T2DM decreased 0.04 (95% CI: 0.01, 0.07, *p* = 0.010). Through a meta-regression equation represented in a bubble plot, we observed that when the proportion of T2DM subjects reached 10%, the protective effect from dietary zinc intake was nullified (Figure 6). Consistently, the three studies with a proportion of T2DM subjects of 10% or more, did not find a significant association between dietary zinc intake and T2DM [6,10,11]. In addition, stratified analysis based on the percentage of T2DM in each study (<5%, 5%–9.9%, and ≥10%) showed an undetectable heterogeneity in all the three subgroups (I^2^ = 0.0%, *p* > 0.100), which provides high reliability to the results. Furthermore, a significant inverse association was found between intake of zinc from diet and T2DM when the percentage of T2DM was lower than 10%, and with the highest effect size when that was less than 5% (Figure 5). It should be noted that the studies with less proportion of T2DM subjects (<5%) were those carried out in rural areas, while those with the highest percentage of diabetics (5%–9.9%, and ≥10%) were the studies conducted in urban areas. In addition, among studies of urban areas, those with a moderate proportion of T2DM (5%–9.9%) retained a significant association between dietary zinc intake and T2DM, although it was more attenuated than those in rural areas. Nevertheless, the studies with higher percentage of T2DM did not find any significant relationship (Figure 5). These results suggest that in rural areas, with less T2DM risk factors, and consequently, less T2DM prevalence, the association between dietary zinc intake and T2DM is significant and the effect size is strong; meanwhile, in urban areas, with a greater exposure to T2DM risk factors and a higher T2DM prevalence, the association is still significant but with a low effect size when T2DM prevalence is moderate, and not significant when the T2DM prevalence is high.

Only two studies have evaluated the effect of a high total zinc intake on the risk of T2DM [4,6], and the overall pooled estimates showed no significant association (Figure 8). The NHS cohort showed a moderate protective effect of total zinc intake, while the MDCS cohort did not find a relationship. Consistent with results from the meta-analysis of the dietary zinc intake, the NHS cohort [4] had a moderate proportion of T2DM (7.3%), meanwhile the MDCS cohort [6] presented the highest percentage of T2DM of all included studies in this systematic review (14.1%). This supports the hypothesis previously raised regarding the impact of the T2DM prevalence on the association between zinc intake and risk of T2DM.

Although, there is currently some evidence of the beneficial effect of zinc supplementation on glycemic control in T2DM patients [9,57], scarce studies support the use of zinc supplements in the prevention of this disease [8]. A recent clinical trial based on zinc supplementation has found a reduction in the progression to T2DM in prediabetic subjects, in addition to an improvement in blood glucose and insulin levels, insulin resistance, and β–cell function [9]. Observational studies that have assessed the association between supplementary zinc intake and risk of T2DM are also scarce [4,6,12]. The overall pooled estimates did not show any significant relationship, neither comparing zinc supplement users versus non-users, nor comparing the highest versus lowest quantile of supplementary zinc intake (Figure 7). Those studies that compared zinc supplement users versus non-users against the risk of T2DM, failed to differentiate between participants who obtained zinc from multivitamin/mineral supplements from those taking individual zinc supplements [6,12]. Thus, a synergistic effect or an interaction between minerals and vitamins supplemented, along with zinc, could have affected the relationship between supplementary zinc intake and the risk of T2DM. Interestingly, the NHS cohort reported a significant inverse association in participants with low dietary zinc intake, but not in those with high dietary zinc intake [4]. In addition, dietary zinc intake was more strongly associated with a lower risk of T2DM among those participants with low zinc intakes from supplements. This seems to suggest that only when the zinc intake is insufficient, zinc supplementation may have benefits. However, when dietary intake is adequate, additional zinc intake from supplementation may not confer further benefit.

To the best of our knowledge, only two cross-sectional studies, conducted within the same population-based HUNT3 study, have evaluated the association between whole blood zinc concentration and T2DM [18,19]. However, the results were dissimilar, likely due to characteristics of participants selected during the sampling. Interestingly, Hansen et al. [18] reported a significant and positive association between whole blood zinc concentration and T2DM, in previously undiagnosed diabetic patients and control subjects [18]. Meanwhile, Simic et al. [19], did not find a significant relationship in previously diagnosed T2DM patients and control subjects. These results seem to suggest that when T2DM is in the early stages, i.e., newly diagnosed, zinc levels are more elevated than non-diabetic subjects, and progressively they are reduced as the disease progresses, which is consistent with our previous systematic review and meta-analysis [20]. That meta-analysis which aimed to compare whole blood zinc concentration between T2DM patients and non-diabetic subjects, showed that duration of T2DM had a relevant influence on concentration of zinc in whole blood [20]. In addition, we found a lower whole blood zinc concentration in T2DM patients; however, this group had, at least, 10.2 ± 8.6 years of duration of diabetes, and differences between cases and controls in that study were not observed [58], in concordance with the study of Simic et al. [19], among previously diagnosed T2DM participants [19].

Since the use of whole blood zinc concentration may be not representative of the total zinc body burden [56], we also assessed the association between zinc and T2DM, through a more reliable biomarker of zinc status, the serum/plasma zinc concentration [59]. We wanted to contrast the hypothesis regarding the impact of the T2DM phases on serum/plasma zinc concentration; however, data were limited to carry out that analysis. Only six studies evaluated this relationship, and results were inconsistent [14,15,16,17,40,41], which was highlighted by high heterogeneity observed after the results were combined. Two of the included studies were responsible for 44.5% of the heterogeneity detected [16,17]. After both studies were excluded, the combined result was more reliable (OR = 1.47, 95% CI: 1.11–1.95; I^2^ = 53.9%, *p* = 0.090). Curiously, these two studies, together with the third that contributed more to the global heterogeneity, were conducted on non-population or community-based studies, i.e., hospital-based settings [16], retired employees of a motor company [15] and postmenopausal women on a voluntary basis [17], so the results could not be extrapolated to the general population. However, the other three studies were carried out on population [14] or community-based [40,41] studies. Stratified analysis according to the “sample base” (Figure 9) showed a high heterogeneity in the “non-population or community-based studies” group (I^2^ = 98.0%, *p* < 0.001), and a low heterogeneity in the “population or community-based studies” group (I^2^ = 22.5%, *p* = 0.275). The pooled estimates for this last subgroup revealed a direct and significant association between serum/plasma zinc concentration and T2DM (OR = 1.64, 95% CI: 1.25–2.14). This finding is not consistent with a previous meta-analysis that compared serum/plasma zinc levels between T2DM patients and healthy controls [60]. Results of this previous meta-analysis showed significantly lower serum/plasma zinc concentration in diabetic subjects compared to healthy controls, but with high heterogeneity. The high heterogeneity suggests that results were influenced by confounding factors, but its source was not analyzed in that meta-analysis. Finally, a recent cross-sectional study reported that urinary zinc levels were positively associated with T2DM [61]. These findings suggest this is a response mechanism against zinc excess in serum/plasma in diabetic patients, and it seems to be in concordance with the direct relationship between serum/plasma and T2DM that we observed in our meta-analysis.

Several limitations in the present systematic review and meta-analysis should be considered. First, the number of results and studies included in meta-analyses was small, and stratified analyses might have insufficient power to identify potential confounding factors, as well as to detect potential sources of heterogeneity. To correct this weakness, random effects meta-regressions were carried out. Furthermore, our findings were likely to be influenced by imprecise measurement of zinc intake. However, VFFQ and VDHQ were used to assess dietary, supplementary, and/or total zinc intake, in all but two studies. In addition, differences in diagnostic criteria for the ascertainment of T2DM over the years could have introduced misclassification bias and could affect results. Finally, meta-analyses were based on observational studies, which are prone to confounding and reverse causation. Nevertheless, for meta-analyses of dietary, supplementary and/or total zinc intake, all but one of the included studies were prospective cohort studies, which allows stronger inferences than cross-sectional studies [62].

Our study has also several strengths. Firstly, the comprehensive and robust search strategy within the framework of the EURRECA Network of Excellence was designed to avoid the loss of relevant studies. Moreover, there were no studies that were excluded for reasons of language, avoiding language bias. In addition, standard tests and visual inspection of funnel plots did not show any evidence for risk of publication bias in any meta-analysis. Furthermore, included studies were of high quality, according to the STROBE Statement [42]. The meta-analyses included 575,851 subjects, had a wide geographical spread, and a diverse ethnicity, giving more validity to the results. Finally, heterogeneity was low or moderate in most of the meta-analyses, which also contributes to the study validity.

The important role of zinc on carbohydrate metabolism via several mechanisms is well established, and this could explain the protective effect of dietary zinc intake on risk of T2DM observed in our meta-analysis. Zinc is involved in synthesis, storage, crystallization, and secretion, as well as the action of insulin and translocation of insulin into the cells [21,22,23,24]. In addition, zinc seems to play a role in insulin sensitivity through the activation of the phosphoinositol-3-kinase/protein kinase B cascade [25]. It has also a role insulin–mimetic, being involved in the regulation process of glucose homeostasis [26]. Moreover, zinc may participate in the suppression of proinflammatory cytokines, such as interleukin-1β [27] and nuclear factor kβ [28], avoiding β-cells’ death and protecting insulin. The underlying mechanism whereby higher serum/plasma and/or whole blood zinc concentration could be related to T2DM is unclear. However, strong evidence supports disturbances in zinc homeostasis associated with T2DM, that could not be linked to zinc status [63,64]. In recent years, it has been proposed that the cellular zinc transport system may play a key role in the pathophysiology of T2DM [65,66]. Thus, differences between diabetic patients and healthy controls in gene expressions for most zinc transporters has been observed [63]. This zinc dyshomeostasis may be caused in the early stages of T2DM, as observed in a trend of increased serum zinc levels from healthy to prediabetic and diabetic postmenopausal women [67].

## 5. Conclusions

Findings from this systematic review and meta-analysis revealed a potential protective effect of a moderately high dietary zinc intake, related to the DRI, on the risk of T2DM. The relationship seems to be stronger and more evident in rural compared to urban areas. In addition, T2DM prevalence may be also a confounding factor for this association, being stronger when the prevalence is low, weak when it is moderate, and disappearing with a high prevalence. Conversely, no associations were observed between total or supplementary zinc intake and T2DM. However, more data are required to explore this relationship more fully.

In addition, an elevated serum/plasma zinc concentration is associated with an increased risk of T2DM in the general population. Meanwhile, high whole blood zinc concentration could be associated with T2DM, likely only at an early phase of the diabetes disease. Additional studies are required to confirm these results, and determine the role of serum/plasma and whole blood zinc concentration in the pathophysiology of T2DM.

## Figures and Tables

**Table 1:** Characteristics of studies reporting the association between dietary zinc intake and risk of type 2 diabetes.

Author, Year (Study)	Location (Area)	Study Design	Follow-Up (Years)	Ethnicity	Gender	Age (Years) in Cases at Baseline (mean ± SD)	Age (Years) in Controls at Baseline (mean ± SD)	Sample Size (T2DM)	T2DM (%)	Ascertainment of T2DM	Zinc Assessment Method	Zinc Intake (mg/day) in Cases (mean ± SD)	Zinc Intake (mg/day) in Controls (mean ± SD)	Effect Size (95% CI)
Drake, 2017 (MDCS)	Sweden (urban)	Prospective cohort	Median: 19	White	Men/Women	58.0 ± 7.0	57.8 ± 7.7	26,132 (3676)	14.1	FPG ≥ 7.0 mmol/L (twice), or registries	VDHQ	11.6 ± 3.6	11.1 ± 3.3	HR: 1.07 (0.88–1.30)
Eshak, 2017 (JACC)	Japan (rural, mostly)	Prospective cohort	5	Japanese	Men/Women	Range: 40–65		16,160 (396)	2.5	Self-report	VFFQ	7.3 ± 0.8		OR: 0.64 (0.54–1.00)
Park, 2016 (CARDIA)	USA (urban)	Prospective cohort	23	African American, Caucasian	Men/Women	Range: 18–30; 27.03 ± 3.61		3960 (418)	10.6	FPG ≥ 7.0 mmol/L, or 2-h 75-g OGTT ≥ 11.1 mmol/L, or HbA1c ≥ 6.5%, or drugs	VDHQ	16.7		HR: 1.27 (0.81–2.01)
Vashum, 2013 (ALSWH)	Australia (rural, mostly)	Prospective cohort	6	Australian	Women	Range: 45–50		8921 (333)	3.7	Self-report	VFFQ	10.7		OR: 0.50 (0.32–0.77)
de Oliveira Otto, 2012 (MESA)	USA (urban)	Prospective cohort	Mean: 4.8	White, Asian, African American, Hispanic	Men/Women	Range: 45–84 61.8 ± 10.3		4982 (499)	10.0	FPG ≥ 6.99 mmol/L, or self-reported, or drugs	VFFQ	Median (standard error) 8.3 (4.4)		HR: 1.41 (0.88–2.27)
Sun, 2009 (NHS)	USA (urban)	Prospective cohort	24	White	Women	Range: 33–60		82,297 (6030)	7.3	Self-report	VFFQ	N/A	N/A	RR: 0.92 (0.84–1.00)
Singh, 1998	India (rural)	Cross-sectional study	N/A	South Asian	Men	25–64		894 (27)	3.0	FPG > 7.7 mmol/L, or 2-h 75-g OGTT > 11.1 mmol/L	7-day dietary record	8.8 ± 2.2		OR: 0.61 (0.35–1.66)
					Women			875 (24)	2.7			8.1 ± 2.1		OR: 0.58 (0.44–1.15)
	India (urban)				Men			904 (63)	7.0			7.0 ± 2.0		OR: 0.90 (0.82–0.98)
					Women			902 (45)	5.0			5.6 ± 1.6		OR: 0.85 (0.71–0.93)

Abbreviations: SD, Standard Deviation; T2DM, Type 2 Diabetes Mellitus; MDCS, Malmö Diet and Cancer Study; FPG, Fasting Plasma Glucose; VDHQ, Validated Diet History Questionnaire; HR, Hazard Ratio; CI, Confidence Interval; JACC, Japan Collaborative Cohort; VFFQ, Validated Food Frequency Questionnaire; OR, Odds Ratio; CARDIA, Coronary Artery Risk Development in Young Adults; OGTT, Oral Glucose Tolerance Test; HbA1c, Glycosylated Hemoglobin; ALSWH, Australian Longitudinal Study on Women’s Health; MESA, Multi-Ethnic Study of Atherosclerosis; NHS, Nurses’ Health Study; N/A, Not Applicable or Not Available; RR, Relative Risk.

**Table 2:** Characteristics of studies reporting the association between supplementary and total zinc intake and risk of type 2 diabetes.

Author, Year (Study)	Location (Area)	Study Design	Follow-Up (Years)	Ethnicity	Gender	Age (Years) in Cases at Baseline (mean ± SD)	Age (Years) in Controls at Baseline (mean ± SD)	Sample Size (T2DM)	T2DM (%)	Ascertainment of T2DM	Zinc Assessment Method	Zinc Intake (mg/day) in Cases (mean ± SD)	Zinc Intake (mg/day) in Controls (mean ± SD)	Effect Size (95% CI)
Supplementary zinc intake														
Drake, 2017 (MDCS)	Sweden (urban)	Prospective cohort	Median: 19	White	Men/Women	58.0 ± 7.0	57.8 ± 7.7	26,132 (3676)	14.1	FPG ≥ 7.0 mmol/L (twice), or registries	VDHQ	12.3% user	17.7% user	HR: 0.83 (0.71–0.98)
Song, 2011 (NIH-AARP)	USA (urban)	Prospective cohort	10	White, mostly	Men/Women	Range: 50–71		232,007 (14,130)	6.1	Self-report	Dietary survey	12.5% user	5.7% user	OR: 1.05 (0.98–1.13)
Sun, 2009 (NHS)	USA (urban)	Prospective cohort	24	White	Women with low dietary zinc intake	Range: 33–60		27,432 (2002)	7.3	Self-report	VFFQ	6.3% user in 1980–48.6% user in 2004		RR: 0.86 (0.74–0.99)
		Prospective cohort			Women with high dietary zinc intake			27,432 (2002)						RR: 1.05 (0.92–1.19)
Total zinc intake														
Drake, 2017 (MDCS)	Sweden (urban)	Prospective cohort	Median: 19	White	Men/Women	58.0 ± 7.0	57.8 ± 7.7	26,132 (3676)	14.1	FPG ≥ 7.0 mmol/L (twice), or registries	VDHQ	12.9 ± 5.4	13.0 ± 6.2	HR: 1.05 (0.88–1.25)
Sun, 2009 (NHS)	USA (urban)	Prospective cohort	24	White	Women	Range: 33–60	N/A	82,297 (6030)	7.3	Self-report	VFFQ	Median range: 4.9–18.0	N/A	RR: 0.90 (0.82–0.99)

Abbreviations: SD, Standard Deviation; T2DM, Type 2 Diabetes Mellitus; MDCS, Malmö Diet and Cancer Study; FPG, Fasting Plasma Glucose; VDHQ, Validated Diet History Questionnaire; HR, Hazard Ratio; NIH-AARP, National Institutes of Health-American Association of Retired Persons Diet and Health Study; OR, Odds Ratio; NHS, Nurses’ Health Study; N/A, Not Applicable or Not Available; VFFQ, Validated Food Frequency Questionnaire; RR, Relative Risk.

**Table 3:** Characteristics of studies reporting the association between serum/plasma and whole blood zinc concentration and risk of type 2 diabetes.

Author, Year	Location	Study Design	Follow-Up (Years)	Ethnicity	Gender	Age (Years) in Cases at Baseline (mean ± SD)	Age (Years) in Controls at Baseline (mean ± SD)	Sample Size (T2DM)	T2DM (%)	Ascertainment of T2DM	Zinc Assessment Method	Zinc Levels (µg/dL) in Diabetic Subjects (mean ± SD)	Zinc Levels (µg/dL) in Controls (mean ± SD)	Effect Size (95% CI)
Serum/plasma zinc concentration														
Yuan, 2018 (DFTJ)	China (urban)	Nested case-control	4.6	Chinese	Men/Women	62.8 ± 7.2	62.9 ± 7.3	2078 (1039)	N/A	FPG ≥ 7.0 mmoL/L, or HbA1c ≥ 6.5%, or self-reported, or drugs	ICP-MS	169.6 ± 142.4	156.1 ± 126.5	OR: 1.09 (0.81–1.48)
Li, 2017	China (urban)	Cross-sectional	N/A	Chinese Han	Men/Women	Range: 40–92, mean: 66.3	Range: 40–92, mean: 66.5	551 (122)	N/A	RPG ≥ 11.1 mmol/L and symptoms, 2-h OGTT ≥ 11.1 mmol/L, or FPG ≥ 7.0 mmol/L, or HbA1c ≥ 6.5%	ICP-MS	Median: 63.4	Median: 57.5	OR: 2.26 (1.29–3.98)
Zhang, 2017 (REACTION)	China (urban)	Cross-sectional	N/A	Chinese	Men/Women	57.7 ± 7.4	55.2 ± 7.9	1837 (510)	N/A	Self-reported, or FPG > 7.0 mmol/L, or 2-h 75-g OGTT > 11.1 mmol/L	ICP-MS	109.0 ± 26.0	105.0 ± 25.0	OR: 1.79 (1.13–2.84)
Yary, 2016 (KIHD)	Finland (rural / urban)	Prospective cohort	20	N/A	Men	Range: 42–60		2220 (416)	18.7	Self-reported, or FPG ≥ 7.0 mmol/L or 2-h OGTT ≥ 11.1 mmol/L	AAS	95.0 ± 10.0	93.0 ± 12.0	HR: 1.39 (1.04–1.85)
Skalnaya, 2016	Russia (urban)	Cross-sectional	N/A	N/A	Women	55.8 ± 5.3	56.7 ± 6.1	128 (64)	N/A	HbA1c≥6.5%	ICP-MS	96.0 ± 0.2	105.0 ± 0.2	OR: 0.33 (0.14–0.76)
Shan, 2014	China (urban)	Cross-sectional	N/A	Chinese Han	Men/Women	51.0 ± 10.8	42.5 ± 11.6	1578 (785)	N/A	WHO 1999 criteria	ICP-MS	115.0 ± 45.0	172.5 ± 73.0	OR: 0.09 (0.06–0.13)
Whole blood zinc concentration														
Simic, 2017 (HUNT3)	Norway (rural, mostly)	Cross-sectional	N/A	Caucasian, mostly	Men/Women	65.4 ± 10.6	59.2 ± 12.2	876 (267)	N/A	Self-reported	ICP-MS	Median: 764.3 Range (10–90%): 643.6–893.3	Median: 751.2 Range (10–90%): 623.5–878.2	OR: 1.08 (0.59–1.97)
Hansen, 2017 (HUNT3)	Norway (rural, mostly)	Cross-sectional	N/A	Caucasian, mostly	Men/Women	65.2 ± 10.3	61.4 ± 14.1	883 (128)	N/A	FPG ≥ 7.0 mmol/L and/or 2-h OGTT ≥ 11.1 mmol/L	ICP-MS	Median: 799.0 Range (10–90%): 675.0–881.0	Median: 754.0 Range (10–90%): 628.0–885.0	OR: 2.19 (1.05–4.59)

Abbreviations: SD, Standard Deviation; T2DM, Type 2 Diabetes Mellitus; DFTJ, Dongfeng–Tongji; FPG, Fasting Plasma Glucose; N/A, Not Applicable or Not Available; HbA1c, Glycosylated Hemoglobin; ICP-MS, Inductively Coupled Plasma Mass Spectrometry; OR, Odds Ratio; RPG, Random Plasma Glucose; OGTT, Oral Glucose Tolerance Test; REACTION, Risk Evaluation of cAncers in Chinese diabeTic Individuals: a lONgitudinal; KIHD, Kuopio IschaemicHeart Disease Risk Factor Study; AAS, Atomic Absorption Spectrophotometry; HR, Hazard Ratio; WHO, World Health Organization; HUNT, North-TrØndelag Health.

**Table 4:** Stratified meta-analyses and meta-regressions on the association between dietary zinc intake and risk of type 2 diabetes mellitus.

Subgroup	Studies (n)	Effect Size (95% CI)	Heterogeneity		Meta-Regressions					
			I^2^ (%)	*p*-Value	Regression Coefficients (95% CI)	Standard Error	*p*-Value	Tau^2^	I^2^ Residual (%)	Adjusted R^2^ (%)
Geographic area										
Western (1)	5	0.97 (0.77–1.22)	72.1%	0.006	−0.25 (−0.66; 0.17)	0.18	0.208	0.04	64.05%	6.68%
Eastern (2)	5	0.80 (0.70–0.92)	49.3%	0.096						
Geographic regions										
Oceania (1)	1	0.50 (0.32–0.78)	-	-	0.22 (0.04–0.39)	0.08	0.022	0.02	50.38%	67.68%
Asia (2)	5	0.80 (0.70–0.92)	49.3%	0.096						
America (3)	3	1.10 (0.82–1.47)	57.7%	0.094						
Europe (4)	1	1.07 (0.88–1.30)	-	-						
Area of residence										
Rural (1)	4	0.59 (0.48–0.73)	0.0%	0.843	0.45 (0.16; 0.73)	0.13	0.007	0.00	17.82%	100.0%
Urban (2)	6	0.94 (0.86–1.02)	43.9%	0.113						
Gender										
Men (1)	2	0.90 (0.82–0.98)	0.0%	0.331	0.14 (−0.17; 0.45)	0.13	0.330	0.06	67.67%	−30.54%
Women (2)	4	0.78 (0.65–0.95)	71.4%	0.015						
Men/Women (3)	4	1.02 (0.73–1.42)	74.1%	0.009						
Ethnicity										
White (1)	3	0.86 (0.66–1.12)	79.4%	0.008	0.10 (−0.06; 0.26)	0.07	0.194	0.06	68.30%	−17.63%
South Asian (2)	4	0.86 (0.77–0.95)	28.5%	0.241						
Japanese (3)	1	0.64 (0.47–0.87)	-	-						
Several ethnic groups (4)	2	1.34 (0.96–1.85)	0.0%	0.755						
Study design										
Prospective Cohort (1)	6	0.90 (0.73–1.12)	75.1%	0.001	−0.07 (−0.31; 0.16)	0.10	0.495	0.06	67.06%	−33.29%
Cross-sectional (2)	4	0.86 (0.77–0.95)	28.5%	0.241						
Study design and area of residence										
Prospective/Rural (1)	2	0.59 (0.46–0.76)	0.0%	0.367	0.18 (0.08; 0.29)	0.05	0.004	0.004	22.03%	90.47%
Cross-sectional/Rural (2)	2	0.58 (0.39–0.89)	0.0%	0.914						
Cross-sectional/Urban (3)	2	0.88 (0.82–0.95)	0.0%	0.489						
Prospective/Urban (4)	4	1.04 (0.88–1.24)	50.7%	0.107						
Measure of association										
Odds ratio (1)	5	0.75 (0.63–0.88)	63.2%	0.019	0.21 (0.05; 0.39)	0.07	0.016	0.02	49.76%	63.13%
Relative risk (2)	1	0.92 (0.84–1.00)	-	-						
Hazard ratio (3)	4	1.13 (0.96–1.34)	0.0%	0.499						
Sample size										
<1000	4	0.86 (0.77–0.95)	28.5%	0.241	0.03 (−0.09; 0.14)	0.05	0.578	0.06	66.27%	−29.83%
1000–4999	2	1.34 (0.96–1.85)	0.0%	0.755						
5000–9999	2	0.59 (0.46–0.76)	0.0%	0.367						
≥10,000	2	0.97 (0.84–1.11)	47.9%	0.166						
Percentage of T2DM										
<5%	4	0.59 (0.48–0.73)	0.0%	0.843	0.31 (0.15; 0.46)	0.07	0.002	0.00	0.00%	100.0%
5–9.9%	3	0.90 (0.85–0.95)	0.0%	0.627						
≥10%	3	1.13 (0.96–1.34)	0.0%	0.499						
Zinc intake assessment method										
VFFQ (1)	4	0.80 (0.57–1.12)	80.3%	0.002	−0.01 (−0.28; 0.26)	0.12	0.906	0.07	68.24%	−40.61%
VDHQ (2)	2	1.10 (0.92–1.31)	0.0%	0.497						
7-day dietary record (3)	4	0.86 (0.77–0.95)	28.5%	0.241						
Zinc quantiles adjusted for energy										
Adjusted (1)	9	0.86 (0.76–0.96)	65.3%	0.003	0.42 (−0.40; 1.25)	0.36	0.271	0.04	65.34%	6.11%
Not adjusted (2)	1	1.27 (0.81–2.00)	-	-						
Ascertainment of T2DM										
FPG/OGTT (1)	4	0.86 (0.77–0.95)	28.5%	0.241	0.19 (−0.05; 0.43)	0.10	0.112	0.04	62.01%	11.25%
Self-reported (2)	3	0.70 (0.48–1.01)	82.6%	0.003						
Several criteria (3)	3	1.13 (0.96–1.34)	0.0%	0.499						
Diagnostic pattern										
One diagnostic pattern	7	0.81 (0.72–0.91)	61.7%	0.016	0.41 (0.06; 0.77)	0.15	0.027	0.02	53.12%	51.60%
Several diagnostic pattern	3	1.13 (0.96–1.34)	0.0%	0.499						
Study quality										
80–89	6	0.83 (0.71–0.97)	58.4%	0.035	−0.11 (−0.36; 0.58)	0.21	0.598	0.06	65.99%	−23.10%
≥90	4	0.92 (0.73–1.16)	74.0%	0.009						

Abbreviations: CI, Confidence Interval; VDHQ, Validated Diet History Questionnaire; VFFQ, Validated Food Frequency Questionnaire; T2DM, Type 2 Diabetes Mellitus; FPG, Fasting Plasma Glucose; OGTT, Oral Glucose Tolerance Test.

## Supplementary Materials

The following are available online at https://www.mdpi.com/2072-6643/11/5/1027/s1, Figure S1: Funnel plot of publication biases of studies included in the meta-analysis of the association between dietary zinc intake and T2DM. Each dot stands for an individual study. Figure S2: Funnel plot of publication biases of studies included in the meta-analysis of the association between supplementary zinc intake and T2DM. Each dot stands for an individual study. Figure S3: Funnel plot of publication biases of studies included in the meta-analysis of the association between serum/plasma zinc concentration and T2DM. Each dot stands for an individual study. Table S1: MOOSE Checklist for Meta-analyses of Observational Studies. Table S2: STROBE Statement Checklist. Table S3: Stratified meta-analyses and meta-regressions on the association between serum/plasma zinc concentration and risk of type 2 diabetes mellitus.
