# Supplementary material for: Zinc Intake and Status and Risk of Type 2 Diabetes Mellitus: A Systematic Review and Meta-Analysis
Source: Nutrients. 2019 May 8;11(5):1027. doi: 10.3390/nu11051027 (PMC6567047; doi:10.3390/nu11051027)
Supplement: Supplementary file 1 [file nutrients-11-01027-s001.zip › Figure S1.docx]

**Figure S1**. Funnel plot of publication biases of studies included in the meta-analysis of the association between dietary zinc intake and T2DM. Each dot stands for an individual study.
