# Supplementary material for: Zinc Intake and Status and Risk of Type 2 Diabetes Mellitus: A Systematic Review and Meta-Analysis
Source: Nutrients. 2019 May 8;11(5):1027. doi: 10.3390/nu11051027 (PMC6567047; doi:10.3390/nu11051027)
Supplement: Supplementary file 1 [file nutrients-11-01027-s001.zip › Table S2.docx]

| Table S2. STROBE Statement Checklist |  |  |  |  |  |  |  |  |  |  |  |  |  |  |  |  |
| --- | --- | --- | --- | --- | --- | --- | --- | --- | --- | --- | --- | --- | --- | --- | --- | --- |
| **● Yes ○ no - Not applicable** | **Singh, 1998** | **Sun, 2009** | **Song, 2011** | **de Oliveira Otto, 2012** | **Vashum, 2013** | **Shan, 2014** | **Yary, 2016** | **Park, 2016** | **Skalnaya, 2016** | **Zhang, 2017** | **Hansen, 2017** | **Simic, 2017** | **Eshak, 2017** | **Li, 2017** | **Drake, 2017** | **Yuan, 2018** |
| TITLE AND ABSTRACT |  |  |  |  |  |  |  |  |  |  |  |  |  |  |  |  |
| a) Indicate the study's design with a commonly used term in the title or the abstract. | ● | ● | ● | ○ | ● | ● | ● | ○ | ○ | ● | ● | ● | ● | ● | ● | ● |
| b) Provide in the abstract an informative and balance summary of what was done and what was found. | ● | ● | ● | ● | ● | ● | ● | ● | ● | ● | ● | ● | ● | ● | ● | ● |
| INTRODUCTION |  |  |  |  |  |  |  |  |  |  |  |  |  |  |  |  |
| Background/rationale: Explain the scientific background and rationale for the investigation being reported. | ● | ● | ● | ● | ● | ● | ● | ● | ● | ● | ● | ● | ● | ● | ● | ● |
| Objectives: State specific objectives, including any prespecified hypotheses | ● | ● | ● | ● | ● | ● | ● | ● | ● | ● | ● | ● | ● | ● | ● | ● |
| METHODS |  |  |  |  |  |  |  |  |  |  |  |  |  |  |  |  |
| Study design: Present key elements of study design early in the paper. | ● | ● | ● | ● | ● | ● | ○ | ● | ○ | ● | ● | ● | ● | ● | ● | ● |
| Setting: Describe the setting, locations, and relevant dates, including periods of recruitment, exposure, follow-up, and data collection. | ● | ● | ● | ● | ● | ● | ● | ● | ○ | ● | ● | ● | ● | ● | ● | ● |
| Participants |  |  |  |  |  |  |  |  |  |  |  |  |  |  |  |  |
| a1) Cohort study- Give the eligibility criteria, and the sources and methods of selection of participants. Describe methods of follow-up. | - | ● | ○ | ● | ● | - | - | ● | - | - | - | - | ● | - | ● | - |
| a2) Case-control study- Give the eligibility criteria, and the sources and methods of case ascertainment and control selection. Give the rationale for the choice of cases and controls. | - | - | - | - | - | ● | - | - | - | - | - | - | - | - | - | ● |
| a3) Cross-sectional study- Give the eligibility criteria, and the sources and methods of selection of participants. | ● | - | - | - | - | - | ○ | - | ● | ● | ● | ● | - | ● | - | - |
| b1) Cohort study- For matched studies, give matching criteria and number of exposed and unexposed. | - | - | - | - | - | - | - | - | - | - | - | - | ● | - | - | - |
| b2) Case-control study- For matched studies, give matching criteria and the number of controls per case | - | - | - | - | - | ● | - | - | - | - | - | - | - | - | - | ● |
| Variables: Clearly define all outcomes, exposures, predictores, potencial confounders, and effect modifiers. Give diagnostic criteria, if applicable. | ● | ● | ● | ● | ● | ● | ● | ● | ● | ● | ● | ● | ● | ● | ● | ● |
| Data sources/measurement: For each variable of interest, give sources of data and details of methods of assessment (measurement). Describe comparability of assessment methods if there is more than one group | ● | ● | ● | ● | ● | ● | ● | ● | ● | ● | ● | ● | ● | ● | ● | ● |
| Bias: Describe any efforts to address potencial sources of bias. | ● | ● | ● | ● | ● | ● | ● | ● | ● | ● | ● | ● | ● | ● | ● | ● |
| Study size: Explain how the study size was arrived at. | ● | ● | ● | ● | ● | ● | ● | ● | ○ | ● | ● | ● | ● | ● | ● | ● |
| Quantitative variables: Explain how quantitative variables were handled in the analyses. If applicable, describe which groupings were chosen and why. | ● | ● | ● | ● | ● | ● | ● | ● | ● | ● | ● | ● | ● | ● | ● | ● |
| Statistical methods |  |  |  |  |  |  |  |  |  |  |  |  |  |  |  |  |
| a) Describe all statistical methods, including those used to control for confounding. | ● | ● | ● | ● | ● | ● | ● | ● | ● | ● | ● | ● | ● | ● | ● | ● |
| b) Describe any methods used to examine subgroups and interactions. | ● | ● | ● | ● | ● | ● | ● | ● | ● | ● | ● | ● | ● | ● | ● | ● |
| c) Explain how missing data were addressed. | ○ | ● | ○ | ○ | ○ | ○ | ○ | ● | ○ | ○ | ○ | ● | ● | ● | ● | ○ |
| d1) Cohort study- if applicable, explain how loss to follow-up was addressed. | - | ● | ○ | - | - | - | - | - | - | - | - | - | ○ | - | ● | - |
| d2) Case-control study- If applicable, explain how matching of cases and controls was addressed. | - | - | - | - | - | ○ | - | - | - | - | - | - | - | - | - | ● |
| d3) Cross-sectional study- If applicable, describe anlytical methods taking account of sampling strategy | ● | - | - | - | - | - | ● | - | - | - | - | - | - | - | - | - |
| e) Describe any sensitivity analyses. | ● | ● | ● | ● | ● | ● | ● | ● | ● | ● | ● | ● | ● | ● | ● | ● |
| RESULTS |  |  |  |  |  |  |  |  |  |  |  |  |  |  |  |  |
| Participants |  |  |  |  |  |  |  |  |  |  |  |  |  |  |  |  |
| a) Report numbers of individuals at each stage of study (eg numbers potentially eligible, examined for eligibility, confirmed eligible, included in the study, completing follow-up, and analysed). | ○ | ● | ● | ● | ● | ● | ● | ● | ● | ● | ● | ● | ● | ● | ● | ● |
| b) Give reasons for non-participation at each stage. | - | - | ● | ● | ● | - | - | ● | - | - | ● | ● | ● | - | ● | ● |
| c) Consider use of a flow diagram. | ○ | ○ | ○ | ○ | ○ | ○ | ○ | ○ | ○ | ○ | ● | ● | ○ | ○ | ● | ● |
| Descriptive data |  |  |  |  |  |  |  |  |  |  |  |  |  |  |  |  |
| a) Give characteristics of study participants (eg demographic, clinical, social) and information on exposures and potential confounders. | ● | ● | ● | ● | ● | ● | ● | ● | ○ | ○ | ● | ● | ● | ● | ● | ● |
| b) Indicate number of participants with missing data for each variable of interest | ○ | ● | ● | ○ | ○ | ○ | ○ | ○ | ○ | ○ | ○ | ○ | ○ | ● | ● | ○ |
| c) Cohort study- summarise follow-up time (eg average and total amount). | - | ● | ● | ● | ● | - | - | ● | - | - | - | - | ● | - | ● | - |
| Outcome data |  |  |  |  |  |  |  |  |  |  |  |  |  |  |  |  |
| a) Cohort study- Report numbers of outcome events or summary measures over time. | - | ● | ● | ● | ● | - | - | ● | - | - | - | - | ● | - | ● | - |
| b) Case-control study- Report numbers in each exposure category, or summary measures of exposure. | - | - | - | - | - | ● | - | - | - | - | - | - | - | - | - | ● |
| c) Cross-sectional study- Report numbers of outcome events or summary measures. | ● | - | - | - | - | - | ○ | - | ● | ● | ● | ● | - | ● | - | - |
| Main results |  |  |  |  |  |  |  |  |  |  |  |  |  |  |  |  |
| a) Give unadjusted estimates and, if applicable, confounder-adjusted estimates and their precision (eg, 95% confidence interval). Make clear which confounders were adjusted for and why they were included. | ● | ● | ● | ● | ● | ● | ● | ● | ● | ● | ● | ● | ● | ● | ● | ● |
| b) Report category boundaries when continuous variables were categorized. | ● | ● | ● | ● | ● | ● | ● | ● | ● | ○ | ○ | ○ | ○ | ● | - | ● |
| c) If relevant, consider translating estimates of relative risk into absolute risk for a meaningful time period. | - | - | - | - | - | - | - | - | - | - | - | - | - | - | - | - |
| Other analyses: Report other analyses done- eg analyses of subgroups an interactions, and sensitivity analyses. | ● | ● | ● | ● | ● | ● | ● | ● | ● | ○ | ○ | ○ | ○ | ○ | ● | ● |
| DISCUSSION |  |  |  |  |  |  |  |  |  |  |  |  |  |  |  |  |
| Key results: Summarise key results with reference to study objectives. | ● | ● | ● | ● | ● | ● | ● | ● | ● | ● | ● | ● | ● | ● | ● | ● |
| Limitations: Discuss limitations of the study, taking into account sources of potential bias or imprecision. Discuss both direction and magnitude of any potential bias. | ○ | ● | ● | ● | ● | ● | ● | ● | ○ | ○ | ● | ● | ● | ● | ● | ● |
| Interpretation: Give a cautious overall interpretation of results considering objectives, limitations, multiplicity of analyses, reults from similar studies, and other relevant evidence. | ● | ● | ● | ● | ● | ● | ● | ● | ● | ● | ● | ● | ● | ● | ● | ● |
| Generalisability: Discuss the generalisability (external validity) of the study results. | ● | ● | ● | ● | ● | ● | ● | ● | ● | ○ | ● | ● | ● | ● | ● | ● |
| OTHER INFORMATION |  |  |  |  |  |  |  |  |  |  |  |  |  |  |  |  |
| Funding: Give the source of funding and the role of the funders for the present study and, if applicable, for the original study on which the present article is based. | ● | ● | ● | ● | ● | ● | ● | ● | ● | ● | ● | ● | ● | ● | ● | ● |
| Accomplished | 25 | 30 | 28 | 27 | 28 | 27 | 24 | 28 | 20 | 21 | 26 | 27 | 28 | 27 | 31 | 30 |
| Not accomplished | 5 | 1 | 4 | 4 | 3 | 4 | 6 | 3 | 9 | 8 | 4 | 3 | 5 | 2 | 0 | 2 |
| Total of items | 30 | 31 | 32 | 31 | 31 | 31 | 30 | 31 | 29 | 29 | 30 | 30 | 33 | 29 | 31 | 32 |
| Quality (%) | 83 | 97 | 88 | 87 | 90 | 87 | 80 | 90 | 69 | 72 | 87 | 90 | 85 | 93 | 100 | 94 |
