# Supplementary material for: Zinc Intake and Status and Risk of Type 2 Diabetes Mellitus: A Systematic Review and Meta-Analysis
Source: Nutrients. 2019 May 8;11(5):1027. doi: 10.3390/nu11051027 (PMC6567047; doi:10.3390/nu11051027)
Supplement: Supplementary file 1 [file nutrients-11-01027-s001.zip › Table S3.docx]

| Table S3.Stratified meta-analyses and meta-regressions on the association between serum/plasma zinc concentration and risk of type 2 diabetes mellitus. | | | | | | | | | | |
| --- | --- | --- | --- | --- | --- | --- | --- | --- | --- | --- |
| **Subgroup** | **Studies (n)** | **Effect size (95% CI)** | **Heterogeneity** | | **Univariate meta-regression** | | | | | |
|  |  |  | **I^2^ (%)** | **P-value** | **Regression coefficients (95% CI)** | **Standar error** | **p-value** | **Tau^2^** | **I^2^ residual (%)** | **Adjusted R^2^ (%)** |
| Geographic area | | | | | | | | | | |
| Western (1) | 2 | 0.71 (0.17-2.94) | 90.1% | <0.001 | 0.13 (-3.23; 3.48) | 1.21 | 0.921 | 1.87 | 97.48% | -25.43% |
| Eastern (2) | 4 | 0.79 (0.19-3.36) | 98.0% | <0.001 |  |  |  |  |  |  |
| Area of residence | | | | | | | | | | |
| Urban | 5 | 0.67 (0.19-2.34) | 97.4% | <0.001 | 0.73 (-3.34; 4.81) | 4.47 | 0.643 | 1.77 | 97.36% | -18.73% |
| Mixed | 1 | 1.39 (1.04-1.85) | - | - |  |  |  |  |  |  |
| Gender | | | | | | | | | | |
| Men (1) | 1 | 1.39 (1.04-1.85) | - | - | -0.13 (-2.17; 1.92) | 0.74 | 0.870 | 1.86 | 97.42% | -25.06% |
| Women (2) | 1 | 0.33 (0.14-0.76) | - | - |  |  |  |  |  |  |
| Men/Women (3) | 4 | 0.79 (0.19-3.36) | 98.0% | <0.001 |  |  |  |  |  |  |
| Study design | | | | | | | | | | |
| Prospective Cohort studies (1) | 1 | 1.39 (1.04-1.85) | - | - | -0.46 (-2.41; 1.50) | 0.70 | 0.551 | 1.69 | 97.06% | -13.74% |
| Case-control (Nested) studies (2) | 1 | 1.09 (0.81-1.47) | - | - |  |  |  |  |  |  |
| Cross-sectional studies (3) | 4 | 0.59 (0.10-3.36) | 97.7% | <0.001 |  |  |  |  |  |  |
| Sample base | | | | | | | | | | |
| Population/Community-based (1) | 3 | 1.39 (1.04-1.85) | 22.5% | 0.275 | -1.71 (-3.83; 0.41) | 0.76 | 0.088 | 0.81 | 96.09% | 45.66% |
| Non-population/Community based (2) | 3 | 0.32 (0.05-1.94) | 98.0% | <0.001 |  |  |  |  |  |  |
| Measure of association | | | | | | | | | | |
| Odds ratio (1) | 5 | 0.67 (0.19-2.34) | 97.4% | <0.001 | 0.37 (-1.67; 2.41) | 0.73 | 0.643 | 1.77 | 97.36% | -18.73% |
| Hazard ratio (2) | 1 | 1.39 (1.04-1.85) | - | - |  |  |  |  |  |  |
| Sample size | | | | | | | | | | |
| <1000 | 2 | 0.88 (0.13-5.88) | 92.8% | <0.001 | -0.23 (-3.58; 3.13) | 1.21 | 0.861 | 1.84 | 97.60% | -23.96% |
| 1000-4999 | 4 | 0.70 (0.20-2.43) | 98.0% | <0.001 |  |  |  |  |  |  |
| Zinc serum/plasma assessment method | | | | | | | | | | |
| ICP-MS (1) | 5 | 0.67 (0.19-2.34) | 97.4% | <0.001 | 0.73 (-3.34; 4.81) | 1.47 | 0.643 | 1.77 | 97.36% | -18.73% |
| AAS (2) | 1 | 1.39 (1.04-1.85) | - | - |  |  |  |  |  |  |
| Matched | | | | | | | | | | |
| Matched (1) | 5 | 0.67 (0.19-2.34) | 97.4% | <0.001 | 0.73 (-3.34; 4.81) | 1.47 | 0.643 | 1.77 | 97.36% | -18.73% |
| Not Matched (2) | 1 | 1.39 (1.04-1.85) | - | - |  |  |  |  |  |  |
| Ascertainment of T2DM | | | | | | | | | | |
| FPG/OGTT (1) | 2 | 0.40 (0.02-7.50) | 98.9% | <0.001 | 0.48 (-0.46; 1.41) | 0.34 | 0.229 | 1.23 | 96.04% | 17.40% |
| HbA1c (2) | 1 | 0.33 (0.14-0.76) | - | - |  |  |  |  |  |  |
| Several criteria (3) | 3 | 1.41 (1.00-1.97) | 61.2% | 0.076 |  |  |  |  |  |  |
| Diagnostic pattern | | | | | | | | | | |
| One diagnostic pattern (1) | 3 | 0.40 (0.05-3.47) | 97.7% | <0.001 | 1.25 (-1.39; 3.88) | 0.95 | 0.259 | 1.29 | 95.50% | 13.52% |
| Several diagnostic pattern (2) | 3 | 1.34 (1.04-1.73) | 40.3.% | 0.187 |  |  |  |  |  |  |
| Group with higher zinc levels | | | | | | | | | | |
| Case group (1) | 4 | 1.47 (1.11-1.95) | 53.9% | 0.090 | 2.37 (1.13; 3.60) | 0.44 | 0.006 | 0.15 | 71.12% | 89.72% |
| Control group (2) | 2 | 0.16 (0.05-0.54) | 86.4% | 0.007 |  |  |  |  |  |  |
| Study quality | | | | | | | | | | |
| < 80 | 2 | 0.79 (1.15-4.21) | 91.7% | 0.001 | 0.35 (-1.53; 2.23) | 0.68 | 0.634 | 1.76 | 97.60% | -18.07% |
| 80-89 | 2 | 0.35 (0.02-5.19) | 99.2% | <0.001 |  |  |  |  |  |  |
| ≥ 90 | 2 | 1.51 (0.74-3.07) | 79.9% | 0.026 |  |  |  |  |  |  |

Abbreviations: CI, Confidence Interval; AAS, Atomic Absorption Spectrophotometry; ICP-MS, Inductively Coupled Plasma Mass Spectrometry; FPG, Fasting Plasma Glucose; OGTT, Oral Glucose Tolerance Test.
